# IL12 immune therapy clinical trial review: Novel strategies for avoiding CRS-associated cytokines

**DOI:** 10.3389/fimmu.2022.952231

**Published:** 2022-09-20

**Authors:** Zhiliang Jia, Dristhi Ragoonanan, Kris Michael Mahadeo, Jonathan Gill, Richard Gorlick, Elizabeth Shpal, Shulin Li

**Affiliations:** ^1^ Department of Pediatric Research, University of Texas MD Anderson Cancer Center, Houston, TX, United States; ^2^ Department of Stem Cell Transplantation and Cellular Therapy, University of Texas MD Anderson Cancer Center, Houston, TX, United States

**Keywords:** IL-12, clinical trial, CRS, cytokines, T cells

## Abstract

Interleukin 12 (IL-12) is a naturally occurring cytokine that plays a key role in inducing antitumor immune responses, including induction of antitumor immune memory. Currently, no IL-12-based therapeutic products have been approved for clinical application because of its toxicities. On the basis of this review of clinical trials using primarily wild-type IL-12 and different delivery methods, we conclude that the safe utilization of IL-12 is highly dependent on the tumor-specific localization of IL-12 post administration. In this regard, we have developed a cell membrane-anchored and tumor-targeted IL-12-T (attIL12-T) cell product for avoiding toxicity from both IL-12 and T cells-induced cytokine release syndrome in peripheral tissues. A phase I trial using this product which seeks to avoid systemic toxicity and boost antitumor efficacy is on the horizon. Of note, this product also boosts the impact of CAR-T or TCR-T cell efficacy against solid tumors, providing an alternative approach to utilize CAR-T to overcome tumor resistance.

## IL-12 biology

Human interleukin 12 (IL-12) is a proinflammatory cytokine that is produced by dendritic cells (DCs) and activated phagocytes. It is a disulfide-bonded heterodimeric cytokine. Human IL-12 gene was cloned from an Epstein–Barr virus–transformed cell line. IL-12 activates natural killer (NK) cells, induces interferon gamma (IFNγ) production, and is pivotal to the process of regulating the transition from innate to adaptive immunity (1, 2). In addition to NK cells, IL-12 acts directly on other types of lymphocytes, including natural killer T cells and CD8+ T cells, to promote proliferation and enhance their cytotoxicity (3). Because it activates these lymphocytes, IL-12 is considered a potent anticancer agent that enhances differentiation of T-helper 1 (Th1) and promotes T cell-mediated cytolysis of cancer cells (4) (5). IL-12 can also directly stimulate DCs to produce additional IL-12 and promote antigen presentation (6). Clinical trials that delivered wild-type recombinant human IL-12 (rhIL-12) or the human IL-12 gene to cancer patients began toward the end of the last century (7, 8). IL-12 has also been used to treat HIV and hepatitis virus infections (9–11). Our goal in this review is to analyze and compare the toxicity and safety results of clinical trials of IL-12 delivered *via* different routes and various forms (i.e., protein, gene, cells) to identify a safe way for IL-12 to be used clinically. A secondary goal is to discuss tumor-targeted delivery of IL-12, as accumulating evidence shows that tumor-targeted delivery of IL-12 is critical to triggering the antigen spreading associated with long-term tumor eradication.

## Toxicity of human IL-12 therapy

IL-12-armed T cell therapy was found to stimulate a significant antitumor response that induced regression of tumors established in preclinical mouse models (12, 13). However, the early clinical trials that used systemic administration of rhIL-12 were disappointing because of observed toxicities produced by high production of proinflammatory cytokine (7, 8, 14, 15). The most common adverse events reported in these clinical trials were fever and flu-like symptoms. Other common toxicities included fatigue, nausea, vomiting, diarrhea, and headache. Hepatic toxicities were also frequently observed, including elevated alanine transaminase (ALT) and aspartate transaminase (AST). The most frequently experienced hematologic adverse events were leukopenia, anemia, neutropenia, and thrombocytopenia (16).

The most commonly reported hematologic toxicities of recombinant IL-12 (rIL-12) protein therapy are neutropenia and thrombocytopenia (17). In multiple trials, rhIL-12 treatment significantly affected leukocytes (7). In one trial, after the initial administration of rhIL-12, it was observed that in all the major lymphocyte subsets patients developed transient and profound lymphopenia (18). In other trials, it was observed that the absolute peripheral blood lymphocyte counts fell dramatically within 4 hours after administration of rhIL-12; these low levels persisted for the first 24 hours after treatment, though a gradual increase was observed thereafter, with levels returning to normal at 168 hours (19, 20). Unlike a single or initial rhIL-12 treatment, subsequent rhIL-12 administrations were followed by significant increases in patients’ peripheral blood lymphocyte counts (21). A dose-dependent increase of T cells, B cells, and NK cells was also observed *in vivo* in lymphoma and myeloma patients receiving rhIL-12 therapy after peripheral blood autologous stem cell transplantation (22).

These toxicities are at least partially caused by the induction of toxic cytokines. One study showed that in 21 cancer patients, administration of rhIL-12 once a week increased the expression of IFNγ, even at very low doses (15). In addition to IFNγ, increased expression of several other cytokines, including tumor necrosis factor alpha (TNFα) and IP-10, has been noted (14, 15). In a phase I trial that was to determine both the optimal biological dose and safety of IL-12 given together with trastuzumab, patients with metastatic human epidermal growth factor receptor 2 (HER2)-positive nonhematological malignancies were administered trastuzumab on day 1 of each weekly cycle. At the beginning of week 3, rhIL-12 protein was injected intravenously on days 2 and 5. Elevated serum levels of macrophage inflammatory protein-1α, TNFα, IP-10, and monokines induced by IFNγ were observed in the patients who had a clinical response or stabilized disease (23). In another trial in patients with HER2-positive malignancies, it was found that activation of extracellular signal-regulated kinase in peripheral blood mononuclear cells and levels of IFNγ and several other chemokines (MIP-1α, IL-8, RANTES, IP-10, and MIG) increased in patients who experienced a clinical benefit including complete response, partial response, or stable disease. But these increases were not observed in patients with progressive disease (21).

In the trial referenced above of rhIL-12 in 21 cancer patients, besides IFNγ, TNFα, and IP-10, several other cytokines, including monokine induced by interferon-gamma (MIG), IL-10, and IL-4, were found to be increased in peripheral blood mononuclear cells even when IL-12 was given at very low doses (30 ng/kg) (15). In the trial of rhIL-12 in patients with ovarian cancer or peritoneal cancer, 3-fold or greater increases of IFNγ, TNFα, IL-10, IL-8, and vascular endothelial growth factor (VEGF) in the peritoneal fluid were observed post-rhIL-12 administration. IP-10 levels were increased in 5 of 5 patients (14). On the basis of cytokine response profile examinations, it was suggested that either NK or T cells could mediate IL-12’s effects (14). Experimental results showed that IL-12 induced a greater than 2-fold increase of IFNγ in 16 patients and of the mRNA expression of TNFα in peripheral whole blood in 13 of 21 patients (15). Though the presence of cytokines is beneficial to the antitumor response, a decrease in IFNγ concentration was associated with the nonexistence of toxicity from IL-12 (15, 24). These results suggested that IL-12-mediated cytokine release contributed substantially to its toxicity in clinical trials; among the induced cytokines, IFNγ, IP-10, and TNFα were the major players (25–28).

## Route dependence in IL-12 therapy-associated toxicity

Despite its toxicities, trials have demonstrated benefits of IL-12-based therapies depending on the route of delivery. In a clinical trial that included 42 patients, 32 with non-Hodgkin lymphoma and 10 with Hodgkin lymphoma, rhIL-12 was administered intravenously (*n* = 11) or subcutaneously (*n* = 31) (29). Most toxicities were grade 1 or 2, but grade 3 hepatic toxicity (reversible) was also reported in 3 patients, who required dose reduction. It was also reported that the response rates altered according to IL-12 administration route. Among patients who received intravenous IL-12, 40% had a partial or complete response, while only 7% of patients who received subcutaneous IL-12 had a partial or complete response. These results clearly demonstrated that at doses with similar toxicity profiles, intravenous delivery of IL-12 produced a better response rate than subcutaneous delivery.

To avoid cytokine toxicity following systemic (i.e., intravenous, subcutaneous) delivery of the IL-12 protein, intratumoral delivery of the IL-12 gene has been investigated. In a trial whose results were published in 2007, DNA encoding an IL-12 plasmid was injected into metastatic lesions between 0.5 and 2.5 cm in diameter) cutaneous or subcutaneously (30). The tumors were treated with either 3 or 6 intratumoral injections. Significant decrease in the treated lesion’s size was observed in 5 of the 12 patients. The lack of toxicity from this intratumoral treatment was associated with a lack of detection of IL-12 in serum. No greater than grade 1 local toxicity was observed from any patient in this trial, and only 2 patients were reported to have grade 1 local toxic effects. In other studies, intratumoral delivery of IL-12 with viral vectors, plasmid vectors, DCs, and CAR-Ts all showed no toxicities or only mild ones (31–36).

Intramuscular or intratumoral electroporation delivery of IL-12 has been reported in several clinical trials. There was a phase I trial reported in 2008, 24 patients that had metastatic melanoma received intratumoral IL-12 plasmid *via* electroporation on days 1, day 5, and day 8 during a single 39-day cycle. Minimal systemic toxicity was observed. Posttreatment examination showed notable tumor necrosis and lymphocytic infiltrate. Thus, intratumoral IL-12 DNA administration *via* electroporation is not only safe but also efficacious against melanoma (37). While IL-12 DNA was combined with other reagent and delivered intramuscular, no significant difference of adverse events was observed with or without IL-12 (26). The toxicity and efficacy findings of clinical trials using IL-12 wild-type protein, gene, and armed cells are summarized in **Tables 1**–**3**, respectively.

**Table 1:** Summary of human clinical trials of wild-type IL-12 protein therapy.

Citation	Title	IL-12 product/route	Dose and toxicity grade
*Clin Cancer Res*. 1997;**3**(3): 409-17 (7).	Phase I evaluation of intravenous recombinant human interleukin 12 in patients with advanced malignancies	Protein/i.v.	3-1000 ng/kg 1 death
*Blood* 1997;**90**(7): 2541-8 (24).	Effects of single-dose interleukin-12 exposure on interleukin-12-associated toxicity and interferon-gamma production	Protein/i.v.	3-1000 ng/kg 2 deaths
*Clin Cancer Res*. 1998; **4**(5): 1183-91 (38).	Phase I trial of subcutaneous recombinant human interleukin-12 in patients with advanced renal cell carcinoma	Protein/s.c.	0.1, 0.5, 0.75, 1.0, 1.25, and 1.5 µg/kg on days 1, 8, and 15 of each 28-day cycle Grade 3 fever, grade 4 gastrointestinal toxicity
*Clin Cancer Res.* 1999; **5**(12): 3983-9 (39).	Phase I study of subcutaneously administered recombinant human interleukin 12 in patients with advanced renal cell cancer	Protein/s.c.	0.5, 1.0, and 1.25 µg/kg Grade 2 and 3 leukopenia, grades 3 and 4 fever, grade 3 granulopenia, grade 3 progressive fatigue, grade 3 vomiting and nausea, grade 3 psychoneurotoxicity
*Clin Cancer Res.* 1999; **5**(1): 9-16 (40).	Immunological effects of interleukin 12 administered by bolus intravenous injection to patients with cancer	Protein/i.v.	3-1000 ng/kg Significant lymphopenia
*Blood* 1999; **94**(3): 902-8 (41).	Interleukin-12 therapy of cutaneous T-cell lymphoma induces lesion regression and cytotoxic T-cell responses	Protein/s.c. or i.t.	50, 100, or 300 ng/kg rhIL-12 twice weekly for up to 24 weeks Low-grade fever and headache
*Clin Cancer Res.* 2000; **6**(5): 1678-92 (42).	Phase I trial of twice-weekly intravenous interleukin 12 in patients with metastatic renal cell cancer or malignant melanoma: ability to maintain IFN-gamma induction is associated with clinical response	Protein/i.v.	30–700 ng/kg twice weekly as an i.v. bolus for 6 weeks Grade 3 hemolytic anemia and grade 3 elevation of serum hepatic transaminases
*Clin Exp Immunol.* 2000; **119**(1): 28-37 (15).	Cytokines and soluble cytokine receptor induction after IL-12 administration in cancer patients	Protein/s.c.	30 ng/kg to 1500 ng/kg once a week N/A
*Clin Cancer Res.* 2000; **6**(7): 2661-9 (43).	A dose-escalation and pharmacokinetic study of subcutaneously administered recombinant human interleukin 12 and its biological effects in Japanese patients with advanced malignancies	Protein/s.c.	50 to 300 ng/kg Grade 3 elevation of aminotransferases, grade 3 leukopenia
*J Immunother.* 2001; **24**(1): 91-8 (44).	Agranulocytosis and hemolytic anemia in patients with renal cell cancer treated with interleukin-12	Protein/i.v.	500 or 700 ng/kg Grade 4 neutropenia, grade 3 hemolytic anemia with transient grade 2 leukopenia and grade 1 thrombocytopenia
*Gynecol Oncol.* 2001; **82**(1): 7-10 (45).	Evaluation of recombinant human interleukin-12 in patients with recurrent or refractory ovarian cancer: a gynecologic oncology group study	Protein/i.v.	250 ng/kg Grade 4 myelotoxicity, grade 4 capillary leak syndrome, grade 3 hepatic toxicity, grade 3 or 4 neutropenia, grade 4 pulmonary toxic effect
*J Clin Oncol.* 2001; **19**(18): 3836-47 (46).	Effects of interleukin-12 on the immune response to a multipeptide vaccine for resected metastatic melanoma	Protein/i.d.	30 ng/kg Transient vaccine-related grade 3 toxicity
*Blood* 2002; **99**(1): 67-74 (47).	Phase 1 study of interleukin-12 in combination with rituximab in patients with B-cell non-Hodgkin lymphoma	Protein/s.c.	30 ng/kg to 500 ng/kg twice weekly Grade 3 follicular lymphoma, grade 3 anemia, grade 3 hypercalcemia, grade 3 vomiting, grade 4 neutropenia
*Clin Cancer Res*. 2002; **8**(12): 3686-95 (48).	Phase I study of intraperitoneal recombinant human interleukin 12 in patients with Mullerian carcinoma, gastrointestinal primary malignancies, and mesothelioma	Protein/i.p.	3 to 600 ng/kg Grade 3 transaminase elevation, grade 3 lymphopenia, grade 3 fever
*Clin Cancer Res.* 2002; **8**(11): 3383-93 (18).	Interleukin 12 immunotherapy after autologous stem cell transplantation for hematological malignancies	Protein/i.v.	30, 100, or 250 ng/kg Transient grade 4 neutropenia or leukopenia, grade 3 diarrhea, grade 3 infection
*Cancer Immun.* 2003; **3**: 7. (49)	Two phase I studies of low dose recombinant human IL-12 with Melan-A and influenza peptides in subjects with advanced malignant melanoma	Protein/s.c. or i.v.	10, 30 and 100 ng/kg Therapy was well tolerated, the main adverse event was influenza-like symptoms
*J Clin Oncol.* 2003; **21 (13**): 2564-73 (50).	Phase I trial of concurrent twice-weekly recombinant human interleukin-12 plus low-dose IL-2 in patients with melanoma or renal cell carcinoma	Protein/i.v.	300 to 500 ng/kg Grade 2 to 3 elevation of hepatic ALT or AST, grade 3 to 4 leukopenia or neutropenia, grade 3 anemia
*J Immunother.* 2003; **26**(3): 270-6 (51).	Intraperitoneal fluid neopterin, nitrate, and tryptophan after regional administration of interleukin-12	Protein/i.p.	100 to 1500 ng/kg N/A
*J Clin Oncol.* 2003; **21**(12): 2342-8 (52).	Immunization with Melan-A peptide-pulsed peripheral blood mononuclear cells plus recombinant human interleukin-12 induces clinical activity and T-cell responses in advanced melanoma	Protein/s.c.	4 μg/kg There were no grade 3 or 4 toxicities
*Clin Cancer Res.* 2003; **9**(1): 76-83 (53).	Repeated administrations of interleukin (IL)-12 are associated with persistently elevated plasma levels of IL-10 and declining IFN-gamma, TNF-α, IL-6, and IL-8 responses	Protein/s.c.	0.1, 0.5, 1.0 and 1.25 µg/kg N/A
*Clin Cancer Res*. 2003; **9**(8): 2950-6 (19).	Pharmacokinetics and immunological aspects of a phase Ib study with intratumoral administration of recombinant human interleukin-12 in patients with head and neck squamous cell carcinoma: a decrease of T-bet in peripheral blood mononuclear cells	Protein/i.t.	100 or 300 ng/kg Grade 4 lymphopenia
*J Immunother*. 2004; **27**(6): 452-9 (54).	Vaccination of glioma patients with fusions of dendritic and glioma cells and recombinant human interleukin 12	Protein/s.c.	6.0 to 37.8 µg total Transient grade 1 fever
*Clin Cancer Res*. 2004; **10**(6): 1935-42 (22).	Immunological consequences of interleukin 12 administration after autologous stem cell transplantation	Protein/i.v.	30, 100, or 250 ng/kg Grade 3 liver toxicity
*Clin Cancer Res*. 2004; **10**(8): 2626-35 (20).	Intratumoral administration of recombinant human interleukin 12 in head and neck squamous cell carcinoma patients elicits a T-helper 1 profile in the locoregional lymph nodes	Protein/i.t.	100 or 300 ng/kg Grade 3 fatigue and liver toxicity, grade 3 lymphopenia and metabolic acidosis
*Gynecol Oncol.* 2004; 3: 957-64 (55).	A phase II trial of interleukin-12 in patients with advanced cervical cancer: clinical and immunologic correlates. Eastern Cooperative Oncology Group study E1E96	Protein/i.v.	250 ng/kg Grade 4 leukopenia, anemia, genitourinary, cardiac toxicity, grade 5 hemorrhage
*Clin Cancer Res.* 2004; **10**(16): 5432-8 (29).	Phase II clinical trial of interleukin-12 in patients with relapsed and refractory non-Hodgkin’s lymphoma and Hodgkin’s disease	Protein/i.v. or s.c.	250 and 500 ng/kg Grade 3 hepatic toxicity
*J Clin Oncol.* 2005; **23**(34): 8835-44 (56).	Phase I study of the sequential combination of interleukin-12 and interferon alfa-2b in advanced cancer: evidence for modulation of interferon signaling pathways by interleukin-12	Protein/s.c. and i.v.	100 to 500 ng/kg No IL-12-related dose-limiting toxicities were observed
*Clin Cancer Res*. 2006; **12**(20 Pt 1): 6056-63 (57).	Randomized phase II study of interleukin-12 in combination with rituximab in previously treated non-Hodgkin’s lymphoma patients	Protein/s.c.	1, 8, 15, and 22 and 300 ng/kg Grade 4 fatigue, hyperglycemia, infection, and leukopenia, grade 3 fever, ALT, AST, rash, and neutropenia
*J Am Acad Dermatol.* 2006; **55**(5): 807-13 (58).	A phase II open-label study of recombinant human interleukin-12 in patients with stage IA, IB, or IIA mycosis fungoides	Protein/s.c.	100 ng/kg for 2 weeks; 300 ng/kg thereafter Treatment-related adverse events were generally mild or moderate, 1 patient in partial response died of hemolytic anemia
*Vaccine* 2006; **24**(25): 5311-9 (59).	Safety and immunogenicity of Towne cytomegalovirus vaccine with or without adjuvant recombinant interleukin-12	Protein/s.c.	0.25, 0.5, 1.0, and 2 µg No severe (grade 3 or 4) adverse events related to study drug exposure occurred.
*Blood* 2006; **107**(12): 4650-7 (60).	Activity of subcutaneous interleukin-12 in AIDS-related Kaposi sarcoma	Protein/s.c.	100 to 625 ng/kg Grade 4 anemia, neutropenia, elevated bilirubin; grade 3 elevated transaminase, amylase, headache, skin rash
*Clin Cancer Res*. 2007; **13**(1): 215-22 (61).	Alum with interleukin-12 augments immunity to a melanoma peptide vaccine: correlation with time to relapse in patients with resected high-risk disease	Protein/s.c.	30 or 100 ng/kg Grade 3 colitis and visual changes, grade 3 headache and nausea, grade 3 bloody diarrhea with a colonoscopy showing lymphocytic colitis
*Clin Cancer Res*. 2007; **13**(5): 1503-10 (62).	Long-term idiotype vaccination combined with interleukin-12 (IL-12), or IL-12 and granulocyte macrophage colony-stimulating factor, in early-stage multiple myeloma patients	Protein/s.c.	2 µg Grade 3 myalgia, grade 1 erythema, and grade 2 edema and/or itching
*J Transl Med*. 2007; **5**: 66 (14).	Phase II study of intraperitoneal recombinant interleukin-12 (rhIL-12) in patients with peritoneal carcinomatosis (residual disease < 1 cm) associated with ovarian cancer or primary peritoneal carcinoma	Protein/i.p.	300 ng/kg Grade 4 neutropenia (1), grade 3 fatigue (4), headache (2), myalgia (2), non-neutropenic fever (1), drug fever (1), back pain (1), and dizziness (1)
*Blood* 2007; **110**(13): 4165-71 (63).	Phase 2 study of pegylated liposomal doxorubicin in combination with interleukin-12 for AIDS-related Kaposi sarcoma	Protein/s.c.	300 and 500 ng/kg Grade 4 elevated aminotransferase, leukopenia, neutropenia, and thrombocytopenia; grade 3 anemia, lymphopenia, and hemolysis
*Int J Cancer* 2008; **123**(10): 2354-61 (5).	Intratumoral rhIL-12 administration in head and neck squamous cell carcinoma patients induces B cell activation	Protein/i.t.	100 or 300 ng/kg N/A
*Mol Cancer Ther.* 2009; **8**(11): 2983-91 (21).	A phase I trial of paclitaxel and trastuzumab in combination with interleukin-12 in patients with HER2/neu-expressing malignancies	Protein/i.v. or s.c.	100, 200, or 300 ng/kg Grade 4 asymptomatic neutropenia and leukopenia, grade 3 fatigue, anorexia, neuropathy, arthralgia, leukopenia, and neutropenia
*Leuk Res.* 2009; **33**(11): 1485-9 (64).	Phase II study of interleukin-12 for treatment of plateau phase multiple myeloma (E1A96): a trial of the Eastern Cooperative Oncology Group	Protein/i.v.	250 and 300 ng/kg Grade 4 hematologic and nonhematologic toxicities
*Exp Hematol Oncol.* 2014; **3**(1): 11 (65).	Single low-dose rHuIL-12 safely triggers multilineage hematopoietic and immune-mediated effects	Protein/s.c.	2, 5, 10, 12, 15, and 20 μg Grade 3 lymphopenia, thrombocytopenia, and neutropenia

s.c., subcutaneous; i.v., intravenous; IL, interleukin; ALT, alanine transaminase; AST, aspartate transaminase; IP, intraperitoneal; TNF-α, tumor necrosis factor alpha; HER, human epidermal growth factor receptor.

**Table 2:** Summary of human clinical trials of IL-12 gene therapy.

Citation	Title	IL-12 gene therapy	Dose and toxicity grade
*Gene Ther.* 1998; **5**(4): 481-90 (66).	Vaccination with IL-12 gene-modified autologous melanoma cells: preclinical results and a first clinical phase I study	Armed tumor cells	1 × 10^5^ to 9.6 × 10^6^ autologous cells. Total 4.6 × 10^6^ to 1.6 × 10^7^ No major toxicity
*Hum Gene Ther*. 2001; **12**(6): 671-84 (67).	Interleukin 12 gene therapy of cancer by peritumoral injection of transduced autologous fibroblasts: outcome of a phase I study	Armed fibroblasts/i.v.	Sufficient fibroblasts to secrete an estimated 300, 1000, 3000, and 5000 ng of IL-12 per 24. Mild to moderate pain at the injection site; no treatment-related changes in hematopoietic, hepatic, renal, or cardiac parameters were observed
*J Neurooncol*. 2003; **64**(1-2): 147-54 (33).	Immunogene therapy of recurrent glioblastoma multiforme with a liposomally encapsulated replication-incompetent Semliki forest virus vector carrying the human interleukin-12 gene–a phase I/II clinical protocol	Liposome/Semliki forest virus IL-12	1 × 10^7^ to 1 × 10^9^/m^2^ No grade 3 or 4 toxicity
*J Clin Oncol.* 2004; **22**(8): 1389-97 (56).	Phase I trial of intratumoral injection of an adenovirus encoding interleukin-12 for advanced digestive tumors	Ad IL-12/i.t.	2.5 × 10^10^ to 3 × 10^12^ Grade 3 lymphopenia, nausea, pleural effusion, and intestinal mucosal edema
*Hum Gene Ther*. 2005; **16**(1): 35-48 (34).	Intratumoral injection of DNA encoding human interleukin 12 into patients with metastatic melanoma: clinical efficacy	Naked IL-12 DNA/i.t.	Low (2 mg, P1-3), medium (4 mg, P4-6), and high (10 to 20 mg, P7-9) amounts of total DNA. None of the 9 patients had a presumably therapy-related adverse event higher than WHO grade 1
*Clin Cancer Res*. 2005; **11**(11): 4168-75 (68).	Phase I study of the intratumoral administration of recombinant canarypox viruses expressing B7.1 and interleukin 12 in patients with metastatic melanoma	Pox virus IL-12/i.t.	25 × 10^8^ pfu Toxicity was mild to moderate
*Cancer Res.* 2007; **67**(4): 1842-52 (31).	Targeting HER-2/neu in early breast cancer development using dendritic cells with staged interleukin-12 burst secretion	IL-12 DC/i.v.	Autologous DC1 (10 to 20 × 10^6^ cells per vaccine) CTC grade 1 or 2 toxicity
*Cytotherapy* 2007; **9**(8): 755-70 (35).	Phase I study of tumor Ag-loaded IL-12 secreting semi-mature DC for the treatment of pediatric cancer	Ad IL-12/i.v.	1, 2, 3, 10 × 10^6^ DCs/m^2^ No major side effects
*Cancer Gene Ther*. 2007; **14**(8): 717-23 (30).	Intratumoral injection of IL-12 plasmid DNA–results of a phase I/IB clinical trial	IL-12 DNA/i.t.	50 mcg Grade 4 pulmonary embolism
*J Clin Oncol.* 2008; **26**(36): 5896-903 (37).	Phase I trial of interleukin-12 plasmid electroporation in patients with metastatic melanoma	IL-12 DNA/i.t.	3.8 or 5.8 mg Minimal systemic toxicity
*Gene Ther.* 2010; **17**(3): 360-9 (69).	Phase-I clinical trial of IL-12 plasmid/lipopolymer complexes for the treatment of recurrent ovarian cancer	IL-12 DNA liposome/i.p.	0.6, 3, 12, or 24 mg/m^2^ Low-grade fever and abdominal pain
*PLoS One* 2012; **7**(1): e29231 (70).	Safety and immunogenicity of an HIV-1 gag DNA vaccine with or without IL-12 and/or IL-15 plasmid cytokine adjuvant in healthy, HIV-1 uninfected adults	IL-12 DNA/s.c.	100, 500, or 1500 µg No significant vaccine-related adverse events
*Gynecol Oncol*. 2013; **131**(1): 169-73 (71).	Phase I trial of a formulated IL-12 plasmid in combination with carboplatin and docetaxel chemotherapy in the treatment of platinum-sensitive recurrent ovarian cancer	Liposome IL-12 DNA/i.p.	12 mg/m^2^, 18 mg/m^2^, and 24 mg/m^2^ DNA Grade 3 abdominal pain, cytokine release syndrome, and hypotension
*J Clin Invest*. 2013; **123**(8): 3383-94 (72).	IL-12p70-producing patient DC vaccine elicits Tc1-polarized immunity	IL-12 DC/i.v.	1.5 × 10^7^ DCs per peptide (6 × 10^7^ DCs total), 5 × 10^6^ DCs per peptide (2 × 10^7^ DCs total) N/A
*J Infect Dis*. 2013; **208**(5): 818-29 (73).	Safety and comparative immunogenicity of an HIV-1 DNA vaccine in combination with plasmid interleukin 12 and impact of intramuscular electroporation for delivery	IL-12 DNA/i.m.	1 or 1.5 mg No severe systemic reactogenicity
*Gynecol Oncol.* 2014; **133**(3): 433-8 (74).	A phase II trial of intraperitoneal EGEN-001, an IL-12 plasmid formulated with PEG-PEI-cholesterol lipopolymer in the treatment of persistent or recurrent epithelial ovarian, fallopian tube or primary peritoneal cancer: a gynecologic oncology group study	Liposome IL-12 DNA/i.p.	24 mg/m^2^ Grade 3 nausea and vomiting (2), anemia (1), and decreased lymphocyte count (1)
*PLoS One* 2015; **10**(8): e0134287 (75).	A phase I double blind, placebo-controlled, randomized study of the safety and immunogenicity of electroporated HIV DNA with or without interleukin 12 in prime-boost combinations with an Ad35 HIV vaccine in healthy HIV-seronegative African adults	IL-12 DNA/i.m.	100 μg and 1000 μg No serious adverse events
*J Acquir Immune Defic Syndr.* 2016; **71**(2): 163-71 (76).	the safety and immunogenicity of an interleukin-12-enhanced multiantigen DNA vaccine delivered by electroporation for the treatment of HIV-1 infection	IL-12 DNA/i.m.	50, 250, or 1000 μg Grade 3 or 4 local injection site pain, with or without bruising and/or swelling
*Sci Transl Med.* 2016; **8**(357): 357ra123 (25).	Cytokine-induced memory-like natural killer cells exhibit enhanced responses against myeloid leukemia	IL-12 proactivated NK/i.v.	0.5, 1.0, 10 × 10^6^/kg NK cells No dose-limiting toxicity or graft vs host disease observed
*Gynecol Oncol.* 2017; **147**(2): 283-90 (77).	A phase I trial of intraperitoneal GEN-1, an IL-12 plasmid formulated with PEG-PEI-cholesterol lipopolymer, administered with pegylated liposomal doxorubicin in patients with recurrent or persistent epithelial ovarian, fallopian tube or primary peritoneal cancers: An NRG Oncology/Gynecologic Oncology Group study	Liposome IL-12 DNA/i.p.	PLD 40 mg/m^2^ or 50 mg/m^2^, GEN-1 at 24 mg/m^2^ or 36 mg/m^2^ Grade 3 leukopenia
*PLoS One* 2018; **13**(9): e0202753 (26).	Safety and tolerability of HIV-1 multiantigen pDNA vaccine given with IL-12 plasmid DNA *via* electroporation, boosted with a recombinant vesicular stomatitis virus HIV Gag vaccine in healthy volunteers in a randomized, controlled clinical trial	IL-12 DNA/i.m.	250, 1000, or 1500 μg IL-12 pDNA did not increase pain scores, reactogenicity, or adverse events with the co-administered DNA vaccine

i.v., intravenous; IL, Interleukin; i.t., intratumoral; WHO, World Health Organization; pfu, plaque-forming unit; HER, human epidermal growth factor receptor; DC, dendritic cell; Ag, antigen; i.p., intraperitoneal; HIV, human immunodeficiency virus; i.m., intramuscular; PLD, pegylated liposomal doxorubicin; pDNA, plasmid DNA.

**Table 3:** Summary of clinical trials of targeted IL-12 therapy.

Citation	Title	Tumor-targeted IL-12	Dose and toxicity grade
*Clin Cancer Res.* 2011; **17**(7): 1998-2005 (78).	A phase 1 study of AS1409, a novel antibody-cytokine fusion protein, in patients with malignant melanoma or renal cell carcinoma	Antibody-IL-12/i.v.	15 and 25 µg/kg Grade 3 fatigue and transaminase elevation, grade 4 anemia
*Clin Cancer Res*. 2015; **21**(10): 2278-88 (79).	Tumor-infiltrating lymphocytes genetically engineered with an inducible gene encoding interleukin-12 for the immunotherapy of metastatic melanoma	IL-12-armed TILs/i.v.	0.01 to 0.1 × 10^9^ NFAT.IL12– transduced TILs/1 time Grade 4 prolonged myelosuppression, elevated LFT, creatinine, grade 3 fever, hypoxia, thrombotic microangiopathy, interstitial pneumonitis
*Sci China Life Sci.* 2016; **59**(4): 386-97 (36).	Phase 1 clinical trial demonstrated that MUC1 positive metastatic seminal vesicle cancer can be effectively eradicated by modified Anti-MUC1 chimeric antigen receptor transduced T cells	IL-12-armed CAR-T/i.t.	5 × 10^5^ pSM3-CAR-T cells/1 time Fever, mild headache, muscle pain, nasal congestion, and mild abdominal bloating discomfort, increased eosinophil ratio and counts, and elevated glucose levels
*Clin Cancer Res.* 2019; **25**(1): 99-109 (80).	First-in-human phase I trial of a tumor-targeted cytokine (NHS-IL12) in subjects with metastatic solid tumors	NHS-IL12 protein/s.c.	0.1, 0.5, 1, 2 4, 8, 12 µg/kg Grade 3 increase in ALT (3; 5.1%), AST, and lipase without clinical signs of pancreatitis (1 each; 1.7%); decreased lymphocyte count (5; 8.5%), neutrophil count (4; 6.8%), and WBCs (2; 3.4%); hypokalemia, hyperhidrosis

i.v., intravenous; IL, Interleukin; TIL, tumor infiltrating lymphocyte; NFAT, nuclear factor of activated T cells; LFT, liver function test; MUC, mucin; ALT, alanine transaminase; AST, aspartate transaminase; WBC, white blood cell.

## Tumor-targeted IL-12–armed antibodies in clinical trials

IL-12 can be covalently linked with monoclonal antibodies to create IL-12-armed antibodies. This design combines the high specificity against target antigen of monoclonal antibodies with the antitumor effects of IL-12, resulting in “armed” monoclonal antibodies that deliver IL-12 to tumor cells that have enriched levels of the target antigen. Such IL-12 delivery targeting a specific antigen minimizes IL-12 exposure in normal tissues and results in less toxicity and a better therapeutic index.

AS1409 is an IL-12-armed antibody in which humanized antibody BC1 is covalently linked to IL-12. It is constructed to deliver IL-12 to tumor-associated vasculature. Humanized antibody BC1 targets the ED-B variant of fibronectin. In a phase I clinical trial, 11 patients with melanoma and 2 with renal cell carcinoma received AS1409 at doses of 15 and 25 μg/kg (78). Most of the observed adverse events were grade 2 or lower. These adverse events included chills, pyrexia, vomiting, fatigue, transient liver function abnormalities, and headache. No dose-limiting toxicities were observed at 15 μg/kg weekly. However, 3 patients had dose-limiting toxicities at the 25 μg/kg dose level. One patient had grade 3 fatigue, and 2 patients had grade 3 transaminase elevation, which resolved after study drug discontinuation. At the 25 μg/kg dose, dose-limiting transaminase elevation and grade 3 fatigue were observed. Of the 11 patients, anemia of grade 3 to 4 was observed in 5 and lymphopenia in 7.

Another IL-12-armed antibody IL12-L19 is being investigated. It targets the splicing variant extra domain B of fibronectin. But there is no toxicity data available from this clinic trial (NCT04471987) yet (81, 82).

## Tumor-targeted tumor-infiltrating lymphocytes or CAR-T cells armed with IL-12 in clinical trials

Tumor-infiltrating lymphocytes (TILs) isolated from tumor specimens have been shown to have highly specific cytolytic activity against their autologous tumors, and this activity inspired the development of TIL immunotherapy at the end of the last century (83–85). In 2015, Zhang et al. reported a clinical trial of IL-12 delivered *via* TILs (79). The trial enrolled 33 patients with metastatic melanoma. Patients were treated with autologous TILs in escalating doses. The TILs were transduced with nuclear factor of activated T cells (NFAT.IL12) promoter driving an IL-12 single-chain gene. Only 1 of the 17 patients who received 0.001 to 0.1 × 10^9^ NFAT.IL12–transduced TILs had an objective response. But among patients who received doses between 0.3 and 3 × 10^9^ cells, 10 of 16 experienced clinical responses. No immediate complications were observed after first cell infusion in all patients. Following that toxicity could be observed. High IL-12 and IFNγ serum levels were observed in patients treated with high cell doses, as were clinical adverse events such as high fever, liver dysfunction, and sporadic severe hemodynamic instability. Increased serum ALT and AST were observed in all cohorts, and the incidence grew higher at higher TIL doses. Fever was observed in all patients. The high IL-12 and IFNγ levels in the serum were life threatening. Some patients were transferred to the intensive care unit for management of these events. Grade 3 adverse events were observed in 16 patients, and grade 4 adverse events were observed in 4 patients. This was the first-in-human trial of autologous TILs that carried an inducible IL-12 gene. It was highlighted that cell doses were 10- to 100-fold lower than the conventional TILs. However, multiple and high-grade toxicities were observed in most patients, and these adverse events were attributable to the secreted IL-12.

Though the IL-12-armed TILs produced toxicity, IL-12-armed CAR-T cell therapy seemed to cause limited toxicity. In a phase I clinical trial of IL-12 delivered *via* mucin 1 (MUC1)-targeting CAR-T cells reported in 2016 by You et al., only mild toxic effects were observed (36). In the study, 2 anti-(MUC1) CAR-T cell lines were constructed. One CAR-T cell line, named SM3-CAR, had an SM3 single-chain variable fragment sequence that target MUC1. The other CAR-T cell line, named pSM3-CAR, contained an SM3 single-chain variable fragment sequence that was modified to have higher binding affinity to MUC1. Only the first CAR-T cells, SM3-CAR co-expressed IL-12. These 2 types of CAR-T cells were injected intratumorally into 2 independent metastatic lesions of the only patient enrolled. Adverse events included mild headache, muscle pain, nasal congestion, and mild abdominal bloating discomfort. All adverse effects resolved by day 12 post intratumoral administration. Analysis of the patient’s blood indicated some abnormalities, such as elevated eosinophil ratio and counts and glucose levels, which suggested an inflammatory response or acute response. The results indicating a serum cytokine response were positive, and the reported side effects of the MUC1-targeting CAR-T cell strategy were very mild.

There is a clinical trial (NCT03932565) that is being investigated. Nectin4/FAP (fibroblast activation protein) targeting CAR-T cells that express IL7 and CCL19, or IL12 were created to treat malignant solid tumors. But there is no toxicity data available from this clinic trial yet.

## Exploration of future safe IL-12 clinical studies

Based on published preclinical studies, we can easily predict that multiple tumor-targeted IL-12 therapies will be evaluated in clinical trials. This approach will also solve the thorny issue of intratumoral delivery for inaccessible metastatic tumors. Both tumor-targeted IL-12 protein and gene/cell therapies will be seen in clinical trials in the near future.

One tumor-targeted IL-12 protein therapy, NHS-tethered IL-12, is particularly noteworthy. Two IL-12 heterodimeric molecules were combined with NHS76 antibody to produce a fusion protein, NHS-IL12 immuno-cytokine. Mechanism studies have proven that IL-12 could be targeted to areas with tumor necrosis by the NHS76 antibody through binding histones to free DNA fragments found in these regions. This targeting mechanism resulted in promoted antitumor activity (80). In the phase I clinical trial, NHS-IL12 were administered subcutaneously to 59 patients with metastatic solid tumors. Of these participants, 22 were enrolled in a single ascending-dose cohort and 37 in an every-4-week multiple ascending-dose cohort. Five patients were reported to have durable stable disease. But no tumor responses were observed. Flu-like symptoms, increased ALT/AST, and decreased circulating lymphocyte count were the most frequently observed treatment-related adverse events. Among all treatment-related grade 3 adverse events, only hyperhidrosis was symptomatic. All others were transient.

Regarding tumor-targeted IL-12 gene therapy, there was design to delivers non-secreting IL-12 with tumor-targeted oncolytic adenovirus (Ad-TD-nsIL12) to tumor cells. The design was tested in Syrian hamster models of pancreatic cancer to examine its therapeutic and toxic effects (86). Surprisingly, no toxic side effects were observed after intraperitoneal delivery of Ad-TD-nsIL12 to orthotopic pancreatic tumors model. The peritoneal disseminated pancreatic tumors were cured. and animal survival was significantly enhanced. After intraperitoneal administration of Ad-TDnsIL12 (1 × 10^9^) into hamsters that had peritoneally disseminated SHPC6 pancreatic tumors, liver function was examined by assessing AST, ALT, and ALP levels in the serum on days 1, 3, and 5. All 3 enzymes were found to remain at levels equivalent to those in the PBS-treated control animals. Serum levels of IL-12 remained constant at all the time points examined, as expected.

Another novel tumor-targeted IL-12 gene is a tumor cell surface vimentin-targeted IL-12 (ttIL12). Combined with resection of the primary tumor, ttIL12 transformed tumors immune profile to INFγ ^Hi^CD8^Hi^FOXP3^Low^CD33^Low^ (87). The immune profile transformation inhibited metastasis and increased survival in both mouse tumor model and patient-derived xenograft tumor mouse models. Mice treated with wild-type IL-12 and surgery had shorter overall survival than the control plasmid DNA treatment group. This significant difference was believed to be the result of IL-12 toxicity. However, IL-12 toxicity was not observed in the ttIL12-treated mice.

Despite the success of tumor targeted IL-12 gene therapy in preclinical models, the success of this therapy in clinical trials is questionable because a therapeutic level of IL-12 must accumulate at the tumor site. Such therapeutic levels were achieved in the previously described trial by Zhang et al. using TIL-armed IL-12 therapy (79). However, that trial demonstrated that cytokines secreted by TILs can also induce toxicity when the number of T cells is high. It seems that avoiding IL-12 secretion before T cells accumulate in tumors will be the key to reduce the toxicity. This hypothesis (concept) provided the foundation for our creation of attIL12-T cell therapy, in which IL-12 is anchored to T cells *via* including a transmembrane domain in the C-terminal of ttIL12 (WO2018068008A1) (27).

This hypothesis (concept) has been validated by a recent publication by Zhang et al., in which the authors found that anchoring IL-12 on T cells avoided IL-12 secretion and the associated systemic toxicity (28). This membrane-anchored IL-12 (aIL12) strategy also increased antitumor efficacy, lowered levels of circulating IL-12 and IFNγ, and did not cause body weight loss in patient-derived xenograft models. NFAT-inducible promoter driven aIL12 expression coordinated the expression of aIL12 and T cell activation. These aIL12-T cells were tested in an OT-1 TCR transgenic murine tumor model. Experimental results showed that aIL12 expression induced by NFAT improved the treatment outcome, but no detectable IL-12 or weight loss was observed. It was then tested in a human tumor xenograft mouse model. The aIL12 expression induced by NFAT enhanced antitumor responses through human T cells that co-expressed tumor-specific engineered TCRs. In both mouse models, this NFAT-inducible IL-12 expression construct did not yield detectable IL-12 in serum.

Both ttIL12- and aIL12-T cells reduced toxic cytokine induction in peripheral tissues. This feature is important because CAR-T cells induce severe cytokine release syndrome in 50% of treated patients (88–90), and this percentage could be reduced by attIL12 modification of these T cells. Supporting this hypothesis, attIL12-modified CAR-T cells or TCR-T cells constrained the production of IL12-induced cytokines in tumors and significantly inhibited cytokine release syndrome-associated cytokines in peripheral tissues of both immune deficient human tumor-bearing mice and immune competent mouse tumor-bearing mice (27).

## Conclusion

The above review includes different types of IL-12 clinical studies using rhIL-12 protein, IL-12-modified cells, and IL-12 genes delivered via physical, chemical, or viral vectors. All these approaches have improved safety, but none of them have eliminated the toxic cytokine release in peripheral tissue associated toxicity. To avoid this clinical side effect, we have invented attIL12-T cell therapy, and others have explored NFAT-inducible IL-12 therapy using mouse models. However, these products, attIL12-modified TILs, TCR-T cells, and CAR-T cells, have yet to be evaluated in the clinical setting. The success of such trials would further boost the enthusiasm for T cell therapy. Of note, attIL12-T cells also boosted T cell penetration and induced antigen spreading, and these bundled features may boost the firepower of currently explored CAR-T cell therapies while reducing the risk of adverse effects.

## Author contributions

ZJ draft the manuscript and conducted the literature search. SL revised and prepared the manuscript. All authors contributed to the article and approved the submitted version.

## Funding

Funding support: NIH/NCI, R01 CA120985.

## Conflict of interest

The authors declare that the research was conducted in the absence of any commercial or financial relationships that could be construed as a potential conflict of interest.

## Publisher’s note

All claims expressed in this article are solely those of the authors and do not necessarily represent those of their affiliated organizations, or those of the publisher, the editors and the reviewers. Any product that may be evaluated in this article, or claim that may be made by its manufacturer, is not guaranteed or endorsed by the publisher.

## Abbreviations

ALT, alanine transaminase; AST, aspartate transaminase; att (cell membrane-anchored and tumor-targeted; IL-12, interleukin 12; CAR, chimeric antigen receptor; DC, dendritic cell; IFNγ, interferon gamma; IL-12, interleukin 12; NK, natural killer; rh, recombinant human; TCR, T cell receptor; CRS, cytokine release syndrome.

## References

[1] Kobayashi M Fitz L Ryan M Hewick RM Clark SC Chan S. Identification and purification of natural killer cell stimulatory factor (NKSF), a cytokine with multiple biologic effects on human lymphocytes. J Exp Med (1989) 170(3):827–45. doi: 10.1084/jem.170.3.827 PMC2189443 2504877

[2] Stern AS Podlaski FJ Hulmes JD Pan YC Quinn PM Wolitzky AG. Purification to homogeneity and partial characterization of cytotoxic lymphocyte maturation factor from human b-lymphoblastoid cells. Proc Natl Acad Sci USA (1990) 87(17):6808–12. doi: 10.1073/pnas.87.17.6808 PMC54627 2204066

[3] Aste-Amezaga M D’Andrea A Kubin M Trinchieri G. Cooperation of natural killer cell stimulatory factor/interleukin-12 with other stimuli in the induction of cytokines and cytotoxic cell-associated molecules in human T and NK cells. Cell Immunol (1994) 156(2):480–92. doi: 10.1006/cimm.1994.1192 7912999

[4] Nguyen HM Guz-Montgomery K Saha D. Oncolytic virus encoding a master pro-inflammatory cytokine interleukin 12 in cancer immunotherapy. Cells (2020) 9(2):400–26. doi: 10.3390/cells9020400 PMC7072539 32050597

[5] van Herpen CM van der Voort R van der Laak JA Klasen IS de Graaf AO van Kempen LC. Intratumoral rhIL-12 administration in head and neck squamous cell carcinoma patients induces b cell activation. Int J Cancer (2008) 123(10):2354–61. doi: 10.1002/ijc.23756 18729197

[6] Grohmann U Belladonna ML Bianchi R Orabona C Ayroldi E Fioretti MC. IL-12 acts directly on DC to promote nuclear localization of NF-kappaB and primes DC for IL-12 production. Immunity (1998) 9(3):315–23. doi: 10.1016/S1074-7613(00)80614-7 9768751

[7] Atkins MB Robertson MJ Gordon M Lotze MT DeCoste M DuBois JS. Phase I evaluation of intravenous recombinant human interleukin 12 in patients with advanced malignancies. Clin Cancer Res (1997) 3(3):409–17. 9815699

[8] Cohen J. IL-12 deaths: explanation and a puzzle. Science (1995) 270(5238):908. doi: 10.1126/science.270.5238.908.a 7481785

[9] Zeuzem S Carreno V. Interleukin-12 in the treatment of chronic hepatitis b and c. Antiviral Res (2001) 52(2):181–8. doi: 10.1016/S0166-3542(01)00183-8 11672828

[10] Rigopoulou EI Suri D Chokshi S Mullerova I Rice S Tedder RS. Lamivudine plus interleukin-12 combination therapy in chronic hepatitis b: antiviral and immunological activity. Hepatol (Baltimore Md) (2005) 42(5):1028–36. doi: 10.1002/hep.20888 16250037

[11] Li SS Kochar NK Elizaga M Hay CM Wilson GJ Cohen KW. DNA Priming increases frequency of T-cell responses to a vesicular stomatitis virus HIV vaccine with specific enhancement of CD8(+) T-cell responses by interleukin-12 plasmid DNA. Clin Vaccine immunol: CVI (2017) 24(11):e00263-17. doi: 10.1128/CVI.00263-17 28931520 PMC5674193

[12] Kerkar SP Muranski P Kaiser A Boni A Sanchez-Perez L Yu Z. Tumor-specific CD8+ T cells expressing interleukin-12 eradicate established cancers in lymphodepleted hosts. Cancer Res (2010) 70(17):6725–34. doi: 10.1158/0008-5472.CAN-10-0735 PMC2935308 20647327

[13] Zhang L Kerkar SP Yu Z Zheng Z Yang S Restifo NP. Improving adoptive T cell therapy by targeting and controlling IL-12 expression to the tumor environment. Mol Ther (2011) 19(4):751–9. doi: 10.1038/mt.2010.313 PMC3070103 21285960

[14] Lenzi R Edwards R June C Seiden MV Garcia ME Rosenblum M. Phase II study of intraperitoneal recombinant interleukin-12 (rhIL-12) in patients with peritoneal carcinomatosis (residual disease < 1 cm) associated with ovarian cancer or primary peritoneal carcinoma. J Trans Med (2007) 5:66. doi: 10.1186/1479-5876-5-66 PMC2248163 18076766

[15] Haicheur N Escudier B Dorval T Negrier S De Mulder PH Dupuy JM. Cytokines and soluble cytokine receptor induction after IL-12 administration in cancer patients. Clin Exp Immunol (2000) 119(1):28–37. doi: 10.1046/j.1365-2249.2000.01112.x 10606961 PMC1905550

[16] Lu X. Impact of IL-12 in cancer. Curr Cancer Drug Targets (2017) 17(8):682–97. doi: 10.2174/1568009617666170427102729 28460617

[17] Lasek W Zagozdzon R Jakobisiak M. Interleukin 12: still a promising candidate for tumor immunotherapy? Cancer immunol immunother: CII (2014) 63(5):419–35. doi: 10.1007/s00262-014-1523-1 PMC3994286 24514955

[18] Robertson MJ Pelloso D Abonour R Hromas RA Nelson RP Jr. Wood L. Interleukin 12 immunotherapy after autologous stem cell transplantation for hematological malignancies. Clin Cancer Res (2002) 8(11):3383–93. 12429625

[19] Van Herpen CM Huijbens R Looman M De Vries J Marres H Van De Ven J. Pharmacokinetics and immunological aspects of a phase ib study with intratumoral administration of recombinant human interleukin-12 in patients with head and neck squamous cell carcinoma: a decrease of T-bet in peripheral blood mononuclear cells. Clin Cancer Res (2003) 9(8):2950–6. 12912941

[20] van Herpen CM Looman M Zonneveld M Scharenborg N de Wilde PC van de Locht L. Intratumoral administration of recombinant human interleukin 12 in head and neck squamous cell carcinoma patients elicits a T-helper 1 profile in the locoregional lymph nodes. Clin Cancer Res (2004) 10(8):2626–35. doi: 10.1158/1078-0432.CCR-03-0304 15102664

[21] Bekaii-Saab TS Roda JM Guenterberg KD Ramaswamy B Young DC Ferketich AK. A phase I trial of paclitaxel and trastuzumab in combination with interleukin-12 in patients with HER2/neu-expressing malignancies. Mol Cancer Ther (2009) 8(11):2983–91. doi: 10.1158/1535-7163.MCT-09-0820 PMC2996611 19887543

[22] Pelloso D Cyran K Timmons L Williams BT Robertson MJ. Immunological consequences of interleukin 12 administration after autologous stem cell transplantation. Clin Cancer Res (2004) 10(6):1935–42. doi: 10.1158/1078-0432.CCR-03-1156 15041709

[23] Parihar R Nadella P Lewis A Jensen R De Hoff C Dierksheide JE. A phase I study of interleukin 12 with trastuzumab in patients with human epidermal growth factor receptor-2-overexpressing malignancies: analysis of sustained interferon gamma production in a subset of patients. Clin Cancer Res (2004) 10(15):5027–37. doi: 10.1158/1078-0432.CCR-04-0265 15297404

[24] Leonard JP Sherman ML Fisher GL Buchanan LJ Larsen G Atkins MB. Effects of single-dose interleukin-12 exposure on interleukin-12-associated toxicity and interferon-gamma production. Blood (1997) 90(7):2541–8. 9326219

[25] Romee R Rosario M Berrien-Elliott MM Wagner JA Jewell BA Schappe T. Cytokine-induced memory-like natural killer cells exhibit enhanced responses against myeloid leukemia. Sci Transl Med (2016) 8(357):357ra123. doi: 10.1126/scitranslmed.aaf2341 PMC5436500 27655849

[26] Elizaga ML Li SS Kochar NK Wilson GJ Allen MA Tieu HVN. Safety and tolerability of HIV-1 multiantigen pDNA vaccine given with IL-12 plasmid DNA *via* electroporation, boosted with a recombinant vesicular stomatitis virus HIV gag vaccine in healthy volunteers in a randomized, controlled clinical trial. PloS One (2018) 13(9):e0202753. doi: 10.1371/journal.pone.0202753 30235286 PMC6147413

[27] Hu J Yang Q Zhang W Du H Chen Y Zhao Q. Cell membrane-anchored and tumor-targeted IL-12 (attIL12)-T cell therapy for eliminating large and heterogeneous solid tumors. J Immunother Cancer (2022) 10(1):e003633. doi: 10.1136/jitc-2021-003633 35027427 PMC8762133

[28] Zhang L Davies JS Serna C Yu Z Restifo NP Rosenberg SA. Enhanced efficacy and limited systemic cytokine exposure with membrane-anchored interleukin-12 T-cell therapy in murine tumor models. J Immunother Cancer (2020) 8(1):e000210. doi: 10.1136/jitc-2019-000210 31959727 PMC7057422

[29] Younes A Pro B Robertson MJ Flinn IW Romaguera JE Hagemeister F. Phase II clinical trial of interleukin-12 in patients with relapsed and refractory non-hodgkin’s lymphoma and hodgkin’s disease. Clin Cancer Res (2004) 10(16):5432–8. doi: 10.1158/1078-0432.CCR-04-0540 15328181

[30] Mahvi DM Henry MB Albertini MR Weber S Meredith K Schalch H. Intratumoral injection of IL-12 plasmid DNA–results of a phase I/IB clinical trial. Cancer Gene Ther (2007) 14(8):717–23. doi: 10.1038/sj.cgt.7701064 17557109

[31] Czerniecki BJ Koski GK Koldovsky U Xu S Cohen PA Mick R. Targeting HER-2/neu in early breast cancer development using dendritic cells with staged interleukin-12 burst secretion. Cancer Res (2007) 67(4):1842–52. doi: 10.1158/0008-5472.CAN-06-4038 17293384

[32] Triozzi PL Allen KO Carlisle RR Craig M LoBuglio AF Conry RM. Phase I study of the intratumoral administration of recombinant canarypox viruses expressing B7.1 and interleukin 12 in patients with metastatic melanoma. Clin Cancer Res (2005) 11(11):4168–75. doi: 10.1158/1078-0432.ccr-04-2283 15930353

[33] Ren H Boulikas T Lundstrom K Soling A Warnke PC Rainov NG. Immunogene therapy of recurrent glioblastoma multiforme with a liposomally encapsulated replication-incompetent semliki forest virus vector carrying the human interleukin-12 gene–a phase I/II clinical protocol. J Neuro-Oncol (2003) 64(1-2):147–54. doi: 10.1007/BF02700029 12952295

[34] Heinzerling L Burg G Dummer R Maier T Oberholzer PA Schultz J. Intratumoral injection of DNA encoding human interleukin 12 into patients with metastatic melanoma: clinical efficacy. Hum Gene Ther (2005) 16(1):35–48. doi: 10.1089/hum.2005.16.35 15703487

[35] Dohnal AM Witt V Hugel H Holter W Gadner H Felzmann T. Phase I study of tumor Ag-loaded IL-12 secreting semi-mature DC for the treatment of pediatric cancer. Cytotherapy (2007) 9(8):755–70. doi: 10.1080/14653240701589221 17917887

[36] You F Jiang L Zhang B Lu Q Zhou Q Liao X. Phase 1 clinical trial demonstrated that MUC1 positive metastatic seminal vesicle cancer can be effectively eradicated by modified anti-MUC1 chimeric antigen receptor transduced T cells. Sci China Life Sci (2016) 59(4):386–97. doi: 10.1007/s11427-016-5024-7 26961900

[37] Daud AI DeConti RC Andrews S Urbas P Riker AI Sondak VK. Phase I trial of interleukin-12 plasmid electroporation in patients with metastatic melanoma. J Clin Oncol (2008) 26(36):5896–903. doi: 10.1200/JCO.2007.15.6794 PMC2645111 19029422

[38] Motzer RJ Rakhit A Schwartz LH Olencki T Malone TM Sandstrom K. Phase I trial of subcutaneous recombinant human interleukin-12 in patients with advanced renal cell carcinoma. Clin Cancer Res (1998) 4(5):1183–91. 9607576

[39] Portielje JE Kruit WH Schuler M Beck J Lamers CH Stoter G. Phase I study of subcutaneously administered recombinant human interleukin 12 in patients with advanced renal cell cancer. Clin Cancer Res (1999) 5(12):3983–9. 10632329

[40] Robertson MJ Cameron C Atkins MB Gordon MS Lotze MT Sherman ML. Immunological effects of interleukin 12 administered by bolus intravenous injection to patients with cancer. Clin Cancer Res (1999) 5(1):9–16. 9918197

[41] Rook AH Wood GS Yoo EK Elenitsas R Kao DM Sherman ML. Interleukin-12 therapy of cutaneous T-cell lymphoma induces lesion regression and cytotoxic T-cell responses. Blood (1999) 94(3):902–8. doi: 10.1182/blood.V94.3.902.415k23_902_908 10419880

[42] Gollob JA Mier JW Veenstra K McDermott DF Clancy D Clancy M. Phase I trial of twice-weekly intravenous interleukin 12 in patients with metastatic renal cell cancer or malignant melanoma: ability to maintain IFN-gamma induction is associated with clinical response. Clin Cancer Res (2000) 6(5):1678–92. 10815886

[43] Ohno R Yamaguchi Y Toge T Kinouchi T Kotake T Shibata M. A dose-escalation and pharmacokinetic study of subcutaneously administered recombinant human interleukin 12 and its biological effects in Japanese patients with advanced malignancies. Clin Cancer Res (2000) 6(7):2661–9. 10914707

[44] Gollob JA Veenstra KG Mier JW Atkins MB. Agranulocytosis and hemolytic anemia in patients with renal cell cancer treated with interleukin-12. J immunother (Hagerstown Md: 1997) (2001) 24(1):91–8. doi: 10.1097/00002371-200101000-00011 11211153

[45] Hurteau JA Blessing JA DeCesare SL Creasman WT. Evaluation of recombinant human interleukin-12 in patients with recurrent or refractory ovarian cancer: a gynecologic oncology group study. Gynecol Oncol (2001) 82(1):7–10. doi: 10.1006/gyno.2001.6255 11426954

[46] Lee P Wang F Kuniyoshi J Rubio V Stuges T Groshen S. Effects of interleukin-12 on the immune response to a multipeptide vaccine for resected metastatic melanoma. J Clin Oncol (2001) 19(18):3836–47. doi: 10.1200/JCO.2001.19.18.3836 11559721

[47] Ansell SM Witzig TE Kurtin PJ Sloan JA Jelinek DF Howell KG. Phase 1 study of interleukin-12 in combination with rituximab in patients with b-cell non-Hodgkin lymphoma. Blood (2002) 99(1):67–74. doi: 10.1182/blood.V99.1.67 11756154

[48] Lenzi R Rosenblum M Verschraegen C Kudelka AP Kavanagh JJ Hicks ME. Phase I study of intraperitoneal recombinant human interleukin 12 in patients with mullerian carcinoma, gastrointestinal primary malignancies, and mesothelioma. Clin Cancer Res (2002) 8(12):3686–95. 12473577

[49] Cebon J Jager E Shackleton MJ Gibbs P Davis ID Hopkins W. Two phase I studies of low dose recombinant human IL-12 with melan-a and influenza peptides in subjects with advanced malignant melanoma. Cancer Immun (2003) 3:7. 12862418

[50] Gollob JA Veenstra KG Parker RA Mier JW McDermott DF Clancy D. Phase I trial of concurrent twice-weekly recombinant human interleukin-12 plus low-dose IL-2 in patients with melanoma or renal cell carcinoma. J Clin Oncol (2003) 21(13):2564–73. doi: 10.1200/JCO.2003.12.119 12829677

[51] Melichar B Lenzi R Rosenblum M Kudelka AP Kavanagh JJ Melicharova K. Intraperitoneal fluid neopterin, nitrate, and tryptophan after regional administration of interleukin-12. J Immunother (Hagerstown Md: 1997). (2003) 26(3):270–6. doi: 10.1097/00002371-200305000-00010 12806280

[52] Peterson AC Harlin H Gajewski TF. Immunization with melan-a peptide-pulsed peripheral blood mononuclear cells plus recombinant human interleukin-12 induces clinical activity and T-cell responses in advanced melanoma. J Clin Oncol (2003) 21(12):2342–8. doi: 10.1200/JCO.2003.12.144 12805336

[53] Portielje JE Lamers CH Kruit WH Sparreboom A Bolhuis RL Stoter G. Repeated administrations of interleukin (IL)-12 are associated with persistently elevated plasma levels of IL-10 and declining IFN-gamma, tumor necrosis factor-alpha, IL-6, and IL-8 responses. Clin Cancer Res (2003) 9(1):76–83. 12538454

[54] Kikuchi T Akasaki Y Abe T Fukuda T Saotome H Ryan JL. Vaccination of glioma patients with fusions of dendritic and glioma cells and recombinant human interleukin 12. J Immunother (Hagerstown Md: 1997) (2004) 27(6):452–9. doi: 10.1097/00002371-200411000-00005 15534489

[55] Wadler S Levy D Frederickson HL Falkson CI Wang Y Weller E. A phase II trial of interleukin-12 in patients with advanced cervical cancer: clinical and immunologic correlates. Eastern cooperative oncology group study E1E96. Gynecol Oncol (2004) 92(3):957–64. doi: 10.1016/j.ygyno.2003.12.022 14984966

[56] Eisenbeis CF Lesinski GB Anghelina M Parihar R Valentino D Liu J. Phase I study of the sequential combination of interleukin-12 and interferon alfa-2b in advanced cancer: evidence for modulation of interferon signaling pathways by interleukin-12. J Clin Oncol (2005) 23(34):8835–44. doi: 10.1200/JCO.2005.02.1691 16314644

[57] Ansell SM Geyer SM Maurer MJ Kurtin PJ Micallef IN Stella P. Randomized phase II study of interleukin-12 in combination with rituximab in previously treated non-hodgkin’s lymphoma patients. Clin Cancer Res (2006) 12(20 Pt 1):6056–63. doi: 10.1158/1078-0432.CCR-06-1245 17062681

[58] Duvic M Sherman ML Wood GS Kuzel TM Olsen E Foss F. A phase II open-label study of recombinant human interleukin-12 in patients with stage IA, IB, or IIA mycosis fungoides. J Am Acad Dermatol (2006) 55(5):807–13. doi: 10.1016/j.jaad.2006.06.038 17052486

[59] Jacobson MA Sinclair E Bredt B Agrillo L Black D Epling CL. Safety and immunogenicity of towne cytomegalovirus vaccine with or without adjuvant recombinant interleukin-12. Vaccine (2006) 24(25):5311–9. doi: 10.1016/j.vaccine.2006.04.017 16701925

[60] Little RF Pluda JM Wyvill KM Rodriguez-Chavez IR Tosato G Catanzaro AT. Activity of subcutaneous interleukin-12 in AIDS-related kaposi sarcoma. Blood (2006) 107(12):4650–7. doi: 10.1182/blood-2005-11-4455 PMC1475826 16507779

[61] Hamid O Solomon JC Scotland R Garcia M Sian S Ye W. Alum with interleukin-12 augments immunity to a melanoma peptide vaccine: Correlation with time to relapse in patients with resected high-risk disease. Clin Cancer Res (2007) 13(1):215–22. doi: 10.1158/1078-0432.CCR-06-1450 17200357

[62] Hansson L Abdalla AO Moshfegh A Choudhury A Rabbani H Nilsson B. Long-term idiotype vaccination combined with interleukin-12 (IL-12), or IL-12 and granulocyte macrophage colony-stimulating factor, in early-stage multiple myeloma patients. Clin Cancer Res (2007) 13(5):1503–10. doi: 10.1158/1078-0432.CCR-06-1603 17332295

[63] Little RF Aleman K Kumar P Wyvill KM Pluda JM Read-Connole E. Phase 2 study of pegylated liposomal doxorubicin in combination with interleukin-12 for AIDS-related kaposi sarcoma. Blood (2007) 110(13):4165–71. doi: 10.1182/blood-2007-06-097568 PMC2234790 17846226

[64] Lacy MQ Jacobus S Blood EA Kay NE Rajkumar SV Greipp PR. Phase II study of interleukin-12 for treatment of plateau phase multiple myeloma (E1A96): A trial of the Eastern cooperative oncology group. Leukemia Res (2009) 33(11):1485–9. doi: 10.1016/j.leukres.2009.01.020 PMC4070590 19243818

[65] Gokhale MS Vainstein V Tom J Thomas S Lawrence CE Gluzman-Poltorak Z. Single low-dose rHuIL-12 safely triggers multilineage hematopoietic and immune-mediated effects. Exp Hematol Oncol (2014) 3(1):11. doi: 10.1186/2162-3619-3-11 24725395 PMC3991894

[66] Sun Y Jurgovsky K Moller P Alijagic S Dorbic T Georgieva J. Vaccination with IL-12 gene-modified autologous melanoma cells: preclinical results and a first clinical phase I study. Gene Ther (1998) 5(4):481–90. doi: 10.1038/sj.gt.3300619 9614572

[67] Kang WK Park C Yoon HL Kim WS Yoon SS Lee MH. Interleukin 12 gene therapy of cancer by peritumoral injection of transduced autologous fibroblasts: outcome of a phase I study. Hum Gene Ther (2001) 12(6):671–84. doi: 10.1089/104303401300057388 11426466

[68] Sangro B Mazzolini G Ruiz J Herraiz M Quiroga J Herrero I. Phase I trial of intratumoral injection of an adenovirus encoding interleukin-12 for advanced digestive tumors. J Clin Oncol (2004) 22(8):1389–97. doi: 10.1200/JCO.2004.04.059 15084613

[69] Anwer K Barnes MN Fewell J Lewis DH Alvarez RD. Phase-I clinical trial of IL-12 plasmid/lipopolymer complexes for the treatment of recurrent ovarian cancer. Gene Ther (2010) 17(3):360–9. doi: 10.1038/gt.2009.159 20033066

[70] Kalams SA Parker S Jin X Elizaga M Metch B Wang M. Safety and immunogenicity of an HIV-1 gag DNA vaccine with or without IL-12 and/or IL-15 plasmid cytokine adjuvant in healthy, HIV-1 uninfected adults. PloS One (2012) 7(1):e29231. doi: 10.1371/journal.pone.0029231 22242162 PMC3252307

[71] Anwer K Kelly FJ Chu C Fewell JG Lewis D Alvarez RD. Phase I trial of a formulated IL-12 plasmid in combination with carboplatin and docetaxel chemotherapy in the treatment of platinum-sensitive recurrent ovarian cancer. Gynecol Oncol (2013) 131(1):169–73. doi: 10.1016/j.ygyno.2013.07.081 23863356

[72] Carreno BM Becker-Hapak M Huang A Chan M Alyasiry A Lie WR. IL-12p70-producing patient DC vaccine elicits Tc1-polarized immunity. J Clin Invest (2013) 123(8):3383–94. doi: 10.1172/JCI68395 PMC3726168 23867552

[73] Kalams SA Parker SD Elizaga M Metch B Edupuganti S Hural J. Safety and comparative immunogenicity of an HIV-1 DNA vaccine in combination with plasmid interleukin 12 and impact of intramuscular electroporation for delivery. J Infect Dis (2013) 208(5):818–29. doi: 10.1093/infdis/jit236 PMC3733506 23840043

[74] Alvarez RD Sill MW Davidson SA Muller CY Bender DP DeBernardo RL. A phase II trial of intraperitoneal EGEN-001, an IL-12 plasmid formulated with PEG-PEI-cholesterol lipopolymer in the treatment of persistent or recurrent epithelial ovarian, fallopian tube or primary peritoneal cancer: a gynecologic oncology group study. Gynecol Oncol (2014) 133(3):433–8. doi: 10.1016/j.ygyno.2014.03.571 PMC4057915 24708919

[75] Mpendo J Mutua G Nyombayire J Ingabire R Nanvubya A Anzala O. A phase I double blind, placebo-controlled, randomized study of the safety and immunogenicity of electroporated HIV DNA with or without interleukin 12 in prime-boost combinations with an Ad35 HIV vaccine in healthy HIV-seronegative African adults. PloS One (2015) 10(8):e0134287. doi: 10.1371/journal.pone.0134287 26252526 PMC4529153

[76] Jacobson JM Zheng L Wilson CC Tebas P Matining RM Egan MA. The safety and immunogenicity of an interleukin-12-Enhanced multiantigen DNA vaccine delivered by electroporation for the treatment of HIV-1 infection. J Acquir Immune defic Syndr (1999) (2016) 71(2):163–71. doi: 10.1097/QAI.0000000000000830 PMC4712745 26761518

[77] Thaker PH Brady WE Lankes HA Odunsi K Bradley WH Moore KN. A phase I trial of intraperitoneal GEN-1, an IL-12 plasmid formulated with PEG-PEI-cholesterol lipopolymer, administered with pegylated liposomal doxorubicin in patients with recurrent or persistent epithelial ovarian, fallopian tube or primary peritoneal cancers: An NRG Oncology/Gynecologic oncology group study. Gynecol Oncol (2017) 147(2):283–90. doi: 10.1016/j.ygyno.2017.08.001 PMC5704992 28802766

[78] Rudman SM Jameson MB McKeage MJ Savage P Jodrell DI Harries M. A phase 1 study of AS1409, a novel antibody-cytokine fusion protein, in patients with malignant melanoma or renal cell carcinoma. Clin Cancer Res (2011) 17(7):1998–2005. doi: 10.1158/1078-0432.CCR-10-2490 21447719 PMC3071333

[79] Zhang L Morgan RA Beane JD Zheng Z Dudley ME Kassim SH. Tumor-infiltrating lymphocytes genetically engineered with an inducible gene encoding interleukin-12 for the immunotherapy of metastatic melanoma. Clin Cancer Res (2015) 21(10):2278–88. doi: 10.1158/1078-0432.CCR-14-2085 PMC4433819 25695689

[80] Strauss J Heery CR Kim JW Jochems C Donahue RN Montgomery AS. First-in-Human phase I trial of a tumor-targeted cytokine (NHS-IL12) in subjects with metastatic solid tumors. Clin Cancer Res (2019) 25(1):99–109. doi: 10.1158/1078-0432.CCR-18-1512 30131389 PMC6320276

[81] Ziffels B Stringhini M Probst P Fugmann T Sturm T Neri D. Antibody-based delivery of cytokine payloads to carbonic anhydrase IX leads to cancer cures in immunocompetent tumor-bearing mice. Mol Cancer Ther (2019) 18(9):1544–54. doi: 10.1158/1535-7163.MCT-18-1301 PMC6994256 31213507

[82] Ongaro T Matasci M Cazzamalli S Gouyou B De Luca R Neri D. A novel anti-cancer L19-interleukin-12 fusion protein with an optimized peptide linker efficiently localizes *in vivo* at the site of tumors. J Biotechnol (2019) 291:17–25. doi: 10.1016/j.jbiotec.2018.12.004 30586544

[83] Topalian SL Muul LM Solomon D Rosenberg SA. Expansion of human tumor infiltrating lymphocytes for use in immunotherapy trials. J Immunol Methods (1987) 102(1):127–41. doi: 10.1016/S0022-1759(87)80018-2 3305708

[84] Rosenberg SA Packard BS Aebersold PM Solomon D Topalian SL Toy ST. Use of tumor-infiltrating lymphocytes and interleukin-2 in the immunotherapy of patients with metastatic melanoma. A preliminary Rep N Engl J Med (1988) 319(25):1676–80. doi: 10.1056/NEJM198812223192527 3264384

[85] Kradin RL Kurnick JT Lazarus DS Preffer FI Dubinett SM Pinto CE. Tumour-infiltrating lymphocytes and interleukin-2 in treatment of advanced cancer. Lancet (London England) (1989) 1(8638):577–80. doi: 10.1016/S0140-6736(89)91609-7 2564111

[86] Wang P Li X Wang J Gao D Li Y Li H. Re-designing interleukin-12 to enhance its safety and potential as an anti-tumor immunotherapeutic agent. Nat Commun (2017) 8(1):1395. doi: 10.1038/s41467-017-01385-8 29123084 PMC5680234

[87] Zhao Q Hu J Mitra A Cutrera J Zhang W Zhang Z. Tumor-targeted IL-12 combined with tumor resection yields a survival-favorable immune profile. J Immunother Cancer (2019) 7(1):154. doi: 10.1186/s40425-019-0631-z 31208461 PMC6580640

[88] Jensen MC Popplewell L Cooper LJ DiGiusto D Kalos M Ostberg JR. Antitransgene rejection responses contribute to attenuated persistence of adoptively transferred CD20/CD19-specific chimeric antigen receptor redirected T cells in humans. Biol Blood marrow Transplant (2010) 16(9):1245–56. doi: 10.1016/j.bbmt.2010.03.014 PMC3383803 20304086

[89] Wang M Munoz J Goy A Locke FL Jacobson CA Hill BT. KTE-X19 CAR T-cell therapy in relapsed or refractory mantle-cell lymphoma. N Engl J Med (2020) 382(14):1331–42. doi: 10.1056/NEJMoa1914347 PMC7731441 32242358

[90] Schuster SJ Bishop MR Tam CS Waller EK Borchmann P McGuirk JP. Tisagenlecleucel in adult relapsed or refractory diffuse Large b-cell lymphoma. N Engl J Med (2019) 380(1):45–56. doi: 10.1056/NEJMoa1804980 30501490

